# Hyperspectral Video Analysis by Motion and Intensity Preprocessing and Subspace Autoencoding

**DOI:** 10.3389/fchem.2022.818974

**Published:** 2022-03-15

**Authors:** Raffaele Vitale, Cyril Ruckebusch, Ingunn Burud, Harald Martens

**Affiliations:** ^1^ Univ. Lille, CNRS, LASIRE (UMR 8516), Laboratoire Avancé de Spectroscopie pour les Interactions, la Réactivité et l’Environnement, Lille, France; ^2^ Faculty of Science and Technology, Norwegian University of Life Sciences, Oslo, Norway; ^3^ Idletechs AS, Trondheim, Norway; ^4^ Department of Engineering Cybernetics, Norwegian University of Science and Technology, Trondheim, Norway

**Keywords:** hyperspectral videos, motion compensation, IDLE modelling, light scattering, light absorption, extended multiplicative signal correction, on-the-fly processing, BIG measurement DATA

## Abstract

Hyperspectral imaging has recently gained increasing attention from academic and industrial world due to its capability of providing both spatial and physico-chemical information about the investigated objects. While this analytical approach is experiencing a substantial success and diffusion in very disparate scenarios, far less exploited is the possibility of collecting sequences of hyperspectral images over time for monitoring dynamic scenes. This trend is mainly justified by the fact that these so-called hyperspectral *videos* usually result in BIG DATA sets, requiring TBs of computer memory to be both stored and processed. Clearly, standard chemometric techniques do need to be somehow adapted or expanded to be capable of dealing with such massive amounts of information. In addition, hyperspectral video data are often affected by many different sources of variations in sample chemistry (for example, light absorption effects) and sample physics (light scattering effects) as well as by systematic errors (associated, *e*.*g*., to fluctuations in the behaviour of the light source and/or of the camera). Therefore, identifying, disentangling and interpreting all these distinct sources of information represents undoubtedly a challenging task. In view of all these aspects, the present work describes a multivariate hybrid modelling framework for the analysis of hyperspectral videos, which involves spatial, spectral and temporal parametrisations of both known and unknown chemical and physical phenomena underlying complex real-world systems. Such a framework encompasses three different computational steps: 1) motions ongoing within the inspected scene are estimated by optical flow analysis and compensated through IDLE modelling; 2) chemical variations are quantified and separated from physical variations by means of Extended Multiplicative Signal Correction (EMSC); 3) the resulting light scattering and light absorption data are subjected to the On-The-Fly Processing and summarised spectrally, spatially and over time. The developed methodology was here tested on a near-infrared hyperspectral video of a piece of wood undergoing drying. It led to a significant reduction of the size of the original measurements recorded and, at the same time, provided valuable information about systematic variations generated by the phenomena behind the monitored process.

## 1 Introduction

### 1.1 Hyperspectral Videos

In the last decade, hyperspectral imaging has experienced a significant diffusion mainly because of its capability of providing spatial and physico-chemical information about the systems under study - Hugelier et al. (2020). By returning whole spectra for all scanned pixels, in fact, a hyperspectral image permits to map the distribution of the constituents of the investigated samples all over the inspected field of view. For this reason, the applications of this analytical approach have lately dramatically increased in many domains of interest, like medicine, forensics, geoscience, urban and environmental surveillance and fire detection—Fischer and Kakoulli (2006); Chuvieco and Kasischke (2007); Hay et al. (2011); Matikainen and Karila (2011); Elmasry et al. (2012); Lu and Fei (2014); Silva et al. (2017); Khan et al. (2018); Vitale et al. (2020a).

Nonetheless, although hyperspectral imaging devices have become rather common tools in both academic and industrial chemistry laboratories, they are rarely configured so as to collect series of hyperspectral images over time for dynamic scene monitoring. There are two reasons behind this tendency: first of all, finding a reasonable compromise between spatial and spectral resolution and recording rate is not an easy and straightforward task; second, these so-called hyperspectral *videos* often translate into BIG DATA sets that can hardly be coped with by methodologies commonly resorted to for the analysis of individual hyperspectral images—for instance, Principal Component Analysis (PCA), Pearson (1901); Hotelling (1933), Partial Least Squares regression (PLS), Wold et al. (1983); Martens and Næs (1989), Multivariate Curve Resolution-Alternating Least Squares (MCR-ALS), Tauler et al. (1995), and Non-Negative Matrix Factorisation (NNMF), Lawton and Sylvestre (1971); Martens (1979). As an example, one can consider that, when storing hundreds of hyperspectral data arrays, the computer memory load is likely to increase up to the order of magnitude of the TBs. Modern workstations cannot readily handle such massive amounts of information and, therefore, standard chemometric techniques do need to be somehow adapted or extended to be possibly utilised in similar scenarios. Furthermore, hyperspectral video data typically account for various phenomena related to sample physics (*e*.*g*., light scattering) and sample chemistry (light absorbance) and can be significantly affected by many different types of systematic errors (like those associated to nuisance fluctuations of the light source and/or the camera). Thus, identifying, disentangling, modelling and interpreting all these distinct sources of variations remains undoubtedly a challenging task.

### 1.2 Hyperspectral Video Analysis

**FIGURE 1:**
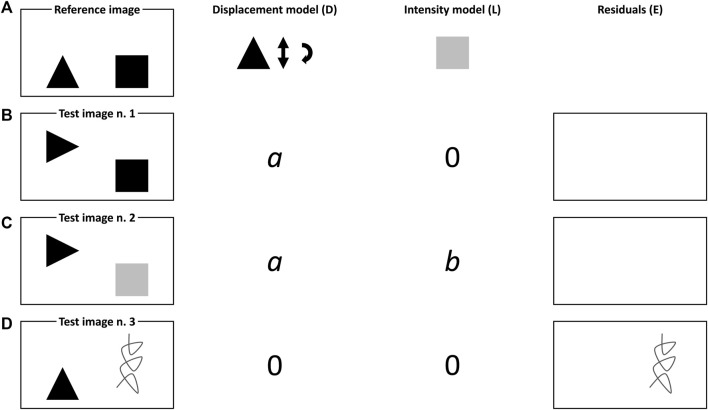
Schematic representation of the basic principles of IDLE modelling. **(A)** Reference image, **(B)** Test image n.1, **(C)** Test image n.2 and **(D)** Test image n.3.

Many known causal phenomena influence how light interacts with matter. The most important ones—light absorbance and light scattering—can even be approximated by relatively simple models, *e*.*g*., the Beer-Lambert’s law or the Kubelka-Munck theory—Bouguer, (1729); Lambert, (1760); Beer (1852); Kubelka and Munk (1931). Yet, albeit some of the aforementioned error factors affecting spectroscopic measurements—like illumination changes—can also be easily foreseen, a detailed mathematical characterisation of the spectral effects they might generate would be prohibitive from a computational point of view. For this reason, modelling and analysing hyperspectral videos constitutes a problematic challenge. Hyperspectral video data, in fact, yield information about the four main ontological aspects of reality: space, time, properties/attributes (for instance, a light intensity profile in the near-infrared—NIR—spectral range) and their interactions. Thus, a comprehensive description of a hyperspectral video would require the identification and the quantification of *factors* or *components* (both known and unknown) accounting for spatial, temporal and spectral variation patterns in such data. This would allow practitioners and users to gain new insights into complex systems of high relevance and into the interplay between the known and unknown phenomena driving their behaviour and evolution. As an immediate example of such an interplay, consider wood drying, a process exhibiting a deep economic and technical impact—McMillen (1964): water absorption properties allow, in principle, the moisture content of wood samples to be accurately determined. However, these properties may substantially change along with the thermodynamic state of water molecules (*i*.*e*., free or bound) and might even mimic spectral contributions from other wood constituents, like cellulose, hemicellulose and lignin. In this as well as in numerous other real-world scenarios, disentangling and characterising the two aforementioned types of phenomena becomes, therefore, crucial from the perspective of understanding. In this article, a novel hybrid approach to achieve this objective is presented. It combines three multivariate approximation strategies for the compression and rational handling of hyperspectral videos: IDLE modelling—Westad and Martens (1999); Martens (2015) — Extended Multiplicative Signal Correction (EMSC)—Martens and Stark (1991); Martens et al. (2003)—and the On-The-Fly Processing (OTFP)—Vitale et al. (2017b). If, on the one hand, EMSC is a well-established tool in the chemometric community, on the other hand IDLE modelling and the OTFP have only recently been conceived, although they have already demonstrated their potential for fast processing of BIG DATA streams—Martens (2015); Vitale et al. (2017b); Stefansson et al. (2020); Vitale et al. (2020b). For the joint analysis of spatial and intensity changes in video recordings, IDLE splits the data variation into two domains as expressed in the following mathematical relation:

$$I=D\left(L\right)+E$$ (1)

by which a generic measured image (I) can be described as a function of the displacement (D) of a local intensity image (L) plus error (E). Imagine, for instance, that two different objects (*i*.*e*., a black triangle and a black square—see Figure 1A) were photographed on a white table. After 1 minute, someone moves the first object along *y*, rotates it 90° and collects another picture (see Figure 1B). After 2 minutes, a third picture is taken after the square was painted grey (see Figure 1C). Assume now that D and L explain simultaneously vertical displacements and clockwise rotations of the black triangle and variations in the black square pixel intensities, respectively. In this simple illustration, D would encode the triangle movement as an individual coefficient (say, *a*), proportional to the dissimilarity from the object’s original location and positioning and exhibiting a sign that depends on the sense of its motion. Analogously, L would quantify the change in the intensity of the pixels of the square as a positive parameter (say, *b*), since the transition from black to light grey implies an increase of the image lightness. Unexpected motions and colours as well as the appearance of unexpected items, like the dark grey spiral-like structure in Figure 1D, would be accounted for by the residuals E.

Conversely, the OTFP gradually constructs reduced-rank bilinear models that summarise virtually *ever*-*lasting* streams of multivariate responses and capture the evolving covariation patterns among their (spectral) variables in space and time. In other words, it represents an extension of classical PCA designed for processing such multivariate responses as soon as they are collected and, most importantly, without requiring entire raw datasets to be kept in memory. More specifically, the OTFP rests on a flexible bilinear subspace model structure which is automatically expanded when a new variation pattern is discovered—as for classical moving-window PCA implementations, Makeig et al. (2000); Wang et al. (2005), even if, here, relevance for *old* or *past* observations is never lost—or refined when the same variation patterns are repeatedly observed, while statistical redundancies are filtered out guaranteeing high rates of information compression. In contrast to black-box *deep learning* solutions, this PCA-like model-based approximation is graphically interpretable in its compressed state and allows at any time the original input to be reconstructed with a better signal-to-noise ratio (as measurement noise is eliminated).

Here, the sequential utilisation of IDLE, EMSC and the OTFP for the investigation of hyperspectral videos will be tested in a context similar to that envisioned before: the monitoring of the drying process of a wood specimen. The results of this study will highlight how this combination can enable an accurate estimation of the dynamic evolution of wood properties and how relatively simple quantitative spatial and temporal information can be extracted from a seemingly overwhelming stream of hyperspectral video data by coupling different mathematical modelling techniques.

### 1.3 Hyperspectral Video Data Structure

Hyperspectral videos can be regarded as time series of three-dimensional data arrays (hyperspectral *frames* or *snapshots*) with dimensions *N*_*x*_ × *N*_*y*_ × *J*, where *N*_*x*_ and *N*_*y*_ denote the number of pixels scanned along the horizontal and vertical direction, respectively, and *J* the number of wavelength channels sampled by the equipment employed. More broadly speaking, they can be thought of as the product of the concatenation of these arrays along a fourth time-related measurement mode. In spite of their multidimensional structure, hyperspectral video data are usually analysed in their unfolded form, *i*.*e*. as matrices of size *N*_*x*_ *N*_*y*_ *K* × *J* with *K* representing the amount of time points at which the aforementioned frames are collected. Each row of such matrices carries a single spectral profile recorded for an individual pixel at a given time point.

## 2 Methods

In this article, EMSC and the OTFP are applied in a sequential fashion to assess/discover and quantify known and unknown sources of data variability in hyperspectral videos. This strategy combines mechanistic and empirical multivariate modelling for describing all physical, chemical and instrumental variation patterns behind hyperspectral video recordings. In order to account for and compensate possible motions and pixel intensity changes which could originate complex non-linearities distorting the measured spatiospectral response, optical flow analysis—Horn and Schunck (1981, 1993)—and IDLE are applied in a preliminary preprocessing step.

The next sections will describe in detail the basics of the three different methodologies exploited here.

### 2.1 IDLE Modelling

Broadly speaking, the IDLE model is a mathematical description of real-world objects or scenes (characterised by spatiotemporal measurements like videos) in terms of their intensity and spatial variations. Here, IDLE is utilised as an empirical compression approach for sets of consecutive video frames, yielding high compression rates and, at the same time, enabling qualitative and quantitative data interpretation. IDLE is based on a three-step methodological procedure:

1. first of all, it segments out each of the relevant, independent objects (so-called *holons*) within a particular scene;
2. then, for each holon it estimates both D (accounting for motions and shape changes) and L, relative to a fixed, user-defined reference frame;
3. finally, it *morphs back* the holons in the investigated image to their spatial shape and location in the reference image. This facilitates a compact subspace modelling of both displacements and intensity changes.

#### 2.1.1 Motion Estimation and Motion Compensation

IDLE modelling concerns how to reduce the complexities that arise when modelling objects that both move and change intensity (or spectral profile) at the same time. Imagine, for instance, a video composed of *K* grey-scale images (**I**_1_, **I**_2_, *…* , **I**_*k*_, *…* , **I**_*K*_) of size *N*_*x*_ × *N*_*y*_ depicting certain objects whose shape and brightness varies over time. Let **I**_ref_ be one of these images, chosen to define a common reference for all the other ones. Analogously, let ${\underline{O}}_{\text{ref}}$ (*N*_*x*_ × *N*_*y*_ × 2) define the horizontal and vertical pixel coordinates (or pixel *addresses*) at which these objects are visible in **I**_ref_. The reference intensity image at pixel adresses ${\underline{O}}_{\text{ref}}$ is then $I_{\text{ref},{\underline{O}}_{\text{ref}}}$ . At this point, the objects in the scene setting captured by each video frame, **I**_*k*_, can be described with respect to how they look in **I**_ref_. Neglecting motions, at time *k*, the local intensity-corrected version of $I_{\text{ref},{\underline{O}}_{\text{ref}}}$ can be expressed as:

$$L_{k,{\underline{O}}_{\text{ref}}}=I_{\text{ref},{\underline{O}}_{\text{ref}}}+\Delta I_{k,{\underline{O}}_{\text{ref}}}$$ (2)

with $\Delta I_{k,{\underline{O}}_{\text{ref}}}$ (*N*_*x*_ × *N*_*y*_) carrying the image intensity deviations from $I_{\text{ref},{\underline{O}}_{\text{ref}}}$ .

Likewise, the pixel adresses where the objects from **I**_ref_ are observable in **I**_*k*_ become:

$${\underline{O}}_{k}={\underline{O}}_{\text{ref}}+\Delta {\underline{O}}_{k}$$ (3)

where $\Delta {\underline{O}}_{k}$ (*N*_*x*_ × *N*_*y*_ × 2) contain the so-called horizontal and vertical motion fields indicating how every pixel in **I**_*k*_ should be displaced so that the objects in **I**_*k*_ mimic their shape and location in **I**_ref_. Hence, merging Eqs (2), (3), the IDLE model for the *k*th frame can be written compactly as a function of how its intensity has changed $\left(\Delta I_{k,{\underline{O}}_{\text{ref}}}\right)$ and how it has moved $\left(\Delta {\underline{O}}_{k}\right)$ compared to the reference one:

$$I_{k,{\underline{O}}_{k}}=I_{k,{\underline{O}}_{\text{ref}}+\Delta {\underline{O}}_{k}}=I_{\text{ref},{\underline{O}}_{\text{ref}}}+\Delta I_{k,{\underline{O}}_{\text{ref}}}=L_{k,{\underline{O}}_{\text{ref}}}$$ (4)

According to this notation, the terms I, D and L in Eq. (1) would correspond to $I_{k,{\underline{O}}_{k}}$ , $\Delta {\underline{O}}_{k}$ and $L_{k,{\underline{O}}_{\text{ref}}}$ , respectively.

From a practical perspective, $\Delta {\underline{O}}_{k}$ can be obtained by motion estimation—Horn and Schunck (1981, 1993)—comparing **I**_*k*_ and **I**_ref_. This allows one to morph the objects from where they were located in **I**_*k*_ back to their pixel addresses in ${\underline{O}}_{\text{ref}}$ and to their intensity at time *k* relative to **I**_ref_ $\left(L_{k,{\underline{O}}_{\text{ref}}}\right)$ . The intensity changes, $\Delta I_{k,{\underline{O}}_{\text{ref}}}$ , as well as the motion fields $\Delta {\underline{O}}_{k}$ are all given in the coordinate system of **I**_ref_, *i*.*e*., ${\underline{O}}_{\text{ref}}$ .

#### 2.1.2 Dual-Domain Bilinear Modelling of a Hyperspectral Video

Even when a hyperspectral video is handled, all the wavelength channels must follow the same spatial displacement at each time *k*. For this purpose, the unfolded vertical and horizontal motion fields, $\Delta o_{k}^{\text{T}}$ (1 × 2*N*_*x*_ *N*_*y*_), can be estimated from an optimised combination of such channels, gathered column-wise into the matrix Δ**O** (*K* × 2*N*_*x*_ *N*_*y*_), modelled bilinearly as

$$\Delta O=T_{\Delta O}P_{\Delta O}^{\text{T}}+E_{\Delta O}^{\text{T}}$$ (5)

and applied to each entire hyperspectral frame. Here, **T**_Δ**O**_ (*K* × *A*_IDLE_) contains the projection coordinates of Δ**O** on the directions defined by the columns of **P**_Δ**O**_ (2*N*_*x*_ *N*_*y*_ × *A*_IDLE_) and **E**_Δ**O**_ (*K* × 2*N*_*x*_ *N*_*y*_) carries the corresponding residuals not explained at the chosen rank, *A*_IDLE_ < 2*N*_*x*_ *N*_*y*_.

Compact, low-dimensional bilinear models often summarize quite well the motions in $\Delta \underline{O}$ when they are defined in the same reference coordinate system. Also the unfolded intensity images $\Delta i_{k,{\underline{O}}_{\text{ref}}}^{\text{T}}$ (1 × *N*_*x*_ *N*_*y*_) may be well approximated in a similar fashion if expressed in a common coordinate system:

$$\Delta I_{{\underline{O}}_{\text{ref}}}=T_{\Delta I_{{\underline{O}}_{\text{ref}}}}P_{\Delta I_{{\underline{O}}_{\text{ref}}}}^{\text{T}}+E_{\Delta I_{{\underline{O}}_{\text{ref}}}}$$ (6)

with $\Delta I_{{\underline{O}}_{\text{ref}}}$ (*K* × *N*_*x*_ *N*_*y*_) being the 2D array resulting from the column-wise concatenation of each $\Delta i_{k,{\underline{O}}_{\text{ref}}}^{\text{T}}$ vector.

Rewriting Eq. (4) in vectorial form, the aforementioned morphing operation can be therefore expressed for the *k*th video frame as:

$$i_{k,{\underline{O}}_{k}}^{\text{T}}=i_{k,{\underline{O}}_{\text{ref}}+\Delta {\underline{O}}_{k}}^{\text{T}}=i_{k,o_{\text{ref}}^{\text{T}}+\Delta o_{k}^{\text{T}}}^{\text{T}}=i_{k,o_{\text{ref}}^{\text{T}}+\left(t_{k,\Delta O}^{\text{T}}P_{\Delta O}^{\text{T}}+e_{k,\Delta O}^{\text{T}}\right)}^{\text{T}}=i_{\text{ref},{\underline{O}}_{\text{ref}}}^{\text{T}}+t_{k,\Delta I_{{\underline{O}}_{\text{ref}}}}^{\text{T}}P_{\Delta I_{{\underline{O}}_{\text{ref}}}}^{\text{T}}+e_{k,\Delta I_{{\underline{O}}_{\text{ref}}}}^{\text{T}}$$ (7)

where $t_{k,\Delta O}^{\text{T}}$ , $e_{k,\Delta O}^{\text{T}}$ , $t_{k,\Delta I_{{\underline{O}}_{\text{ref}}}}^{\text{T}}$ and $e_{k,\Delta I_{{\underline{O}}_{\text{ref}}}}^{\text{T}}$ denote the *k*th row vectors of **T**_Δ**O**_, $E_{\Delta O}^{\text{T}}$ , $T_{\Delta I_{{\underline{O}}_{\text{ref}}}}$ and $E_{\Delta I_{{\underline{O}}_{\text{ref}}}}$ , respectively. Refolding is finally required for the sake of representation.

### 2.2 Extended Multiplicative Signal Correction

EMSC is a bilinear modelling approach that permits to separate, quantify and correct for distinct types of known chemical and physical data variation sources in the acquired signal profiles. As applied in this article, EMSC assumes that a generic spectrum, **x** (of dimensions *J* × 1) can be mathematically described as:

$$x=b\left(r+\underset{i}{\sum }\Delta c_{i}s_{i}\right)+a1+df+gf^{2}+e$$ (8)

where *b* is the effective relative pathlength; **r** (*J* × 1) is a predetermined reference spectrum; Δ*c*_*i*_ and **s**_*i*_ (*J* × 1) denote the presumed concentration/abundance contribution and the spectral fingerprint of the *i*th main constituent of the system under study, respectively; **1** (*J* × 1) is a column vector of ones; **f** (*J* × 1) contains values monotonically increasing from −1 to 1; *a*, *d* and *g* constitute a set of coefficients; and **e** (*J* × 1) carries the unmodelled residuals (*i*.*e*., unmodelled chemical and/or physical variations as well as random measurement noise) resulting from this approximation.

Altogether, **1**, **f** and **f** ^2^ connote polynomial model dimensions accounting for smoothly wavelength-dependent phenomena (baseline level, slope and curvature, respectively).

Given $h_{i}=b\Delta c_{i}\left(\forall i\right)$ , the unknowns in Eq. (8) can be retrieved by Ordinary or Weighted Least Squares (OLS/WLS) as:

$$\left[b\;h_{1}\;\ldots \;h_{I}\;a\;d\;g\right]=x^{\text{T}}W_{\text{EMSC}}W_{\text{EMSC}}M{\left(M^{\text{T}}W_{\text{EMSC}}W_{\text{EMSC}}M\right)}^{-1}$$ (9)

where $M=\left[r\;s_{1}\;\ldots \;s_{I}\;1\;f\;f^{2}\right]$ and **W**_EMSC_ (*J* × *J*) is a diagonal matrix of weights associated to the different sampled spectral channels^1^.

Since the constituent profiles, **s**_*i*_, are a required input for EMSC processing, this methodology has been chosen for describing expected variation patterns evolving all over the duration of a hyperspectral video.

Once the EMSC coefficients have been calculated as in Eq. (9), they can be exploited for pretreating the input spectrum, **x**, in order to filter varying light scattering effects as:

$$x^{\text{p}}=\frac{\left(x-a1-df-gf^{2}\right)}{b}$$ (10)

with ^p^ standing for *preprocessed*. In the present application of EMSC, the estimated chemical variations will also be subtracted from **x** as:

$$x^{\text{p}}=\frac{\left(x-a1-df-gf^{2}-\sum_{i}h_{i}s_{i}\right)}{b}=\frac{\left(x-a1-df-gf^{2}\right)}{b}-\underset{i}{\sum }\Delta c_{i}s_{i}$$ (11)

Finally, if EMSC residuals are deemed to be affected by the effective optical pathlength of the sample, they can be computed as:

$$e=x-br-\underset{i}{\sum }h_{i}s_{i}-a1-df-gf^{2}=x-b\left(r-\underset{i}{\sum }\Delta c_{i}s_{i}\right)-a1-df-gf^{2}$$ (12)

Pathlength-corrected residuals are subsequently estimated as:

$$\tilde{e}=b^{-1}e$$ (13)

### 2.3 The On-The-Fly Processing

After the IDLE-based motion estimation-compensation and the quantification-correction of known physical and chemical variations by EMSC preprocessing, the resulting unmodelled residuals are analysed by the OTFP in the attempt of looking for unknown, yet systematic variability patterns in data. The OTFP relies on a self-learning adaptive modelling principle which allows massive amounts of measurement recordings collected over time to be compressed with a minimal loss of meaningful information according to a PCA-like bilinear decomposition. Its global computational procedure encompasses five different steps:

1. the raw data stream, **X** (of dimensions, *e*.*g*., *N*_*x*_ *N*_*y*_ *K* × *J*), divided into a sequence of blocks, say **X**_*g*_ (*N*_*g*_ × *J*, *g* = 1, 2, *…* , *G*), is submitted to an optional lossless knowledge-based preprocessing stage including a linearisation—which can be conducted by means of approaches like Standard Normal Variate (SNV), Barnes et al. (1989), Multiplicative Scatter Correction (MSC), Martens et al. (1983), Fast Fourier Transform (FFT), Cooley and Tukey (1965), and wavelet decomposition, Walczak (2000)—and a signal-conditioning step;
2. the preprocessed data are projected onto a bilinear subspace already established at the previous point in time as:

$$X_{g}^{\text{p}}=T_{g}^{\text{p}}P^{\text{T}}+E_{g}^{\text{p}}$$ (14)

with $T_{g}^{\text{p}}$ (*N*_*g*_ × *A*_OTFP_) defining the projection coordinates or scores of all the *N*_*g*_ observations on the basis vectors or components defined by the columns of **P** (*J* × *A*_OTFP_) and $E_{g}^{\text{p}}$ (*N*_*g*_ × *J*) carrying unmodelled residuals, *i*.*e*., the fraction of $X_{g}^{\text{p}}$ not explained by the model at the chosen rank, *A*_OTFP_ < *J*;

3. the projection residuals are thereafter input to a second bilinear modelling stage aimed at detecting new components and isolating outliers. New components are encoded as additional subspace dimensions, whose final number is usually selected based on the total amount of the original data variance that is to be explained, although alternative criteria may also be exploited—Endrizzi et al. (2014); Vitale et al. (2017a); Vitale and Saccenti (2018). In other words, the OTFP algorithm learns to identify and quantify all the systematic types of covariation in the data as they stream, while filtering out random measurement errors and irrelevant outliers (if they do not contribute to the definition of a new pattern of variation);
4. at regular intervals, the OTFP model is refined and updated;
5. pretreatment as well as model parameters (*i*.*e*., OTFP scores and loadings) are stored as output. At any time, they can be either used to reconstruct the original data, *e*.*g*., for visualisation, or exploited in their compressed form for efficient storage and transmission, human graphical interpretation and quantification.

A survey of the operational principles of the OTFP is provided in Vitale et al. (2017b).

## 3 Dataset

**FIGURE 2:**
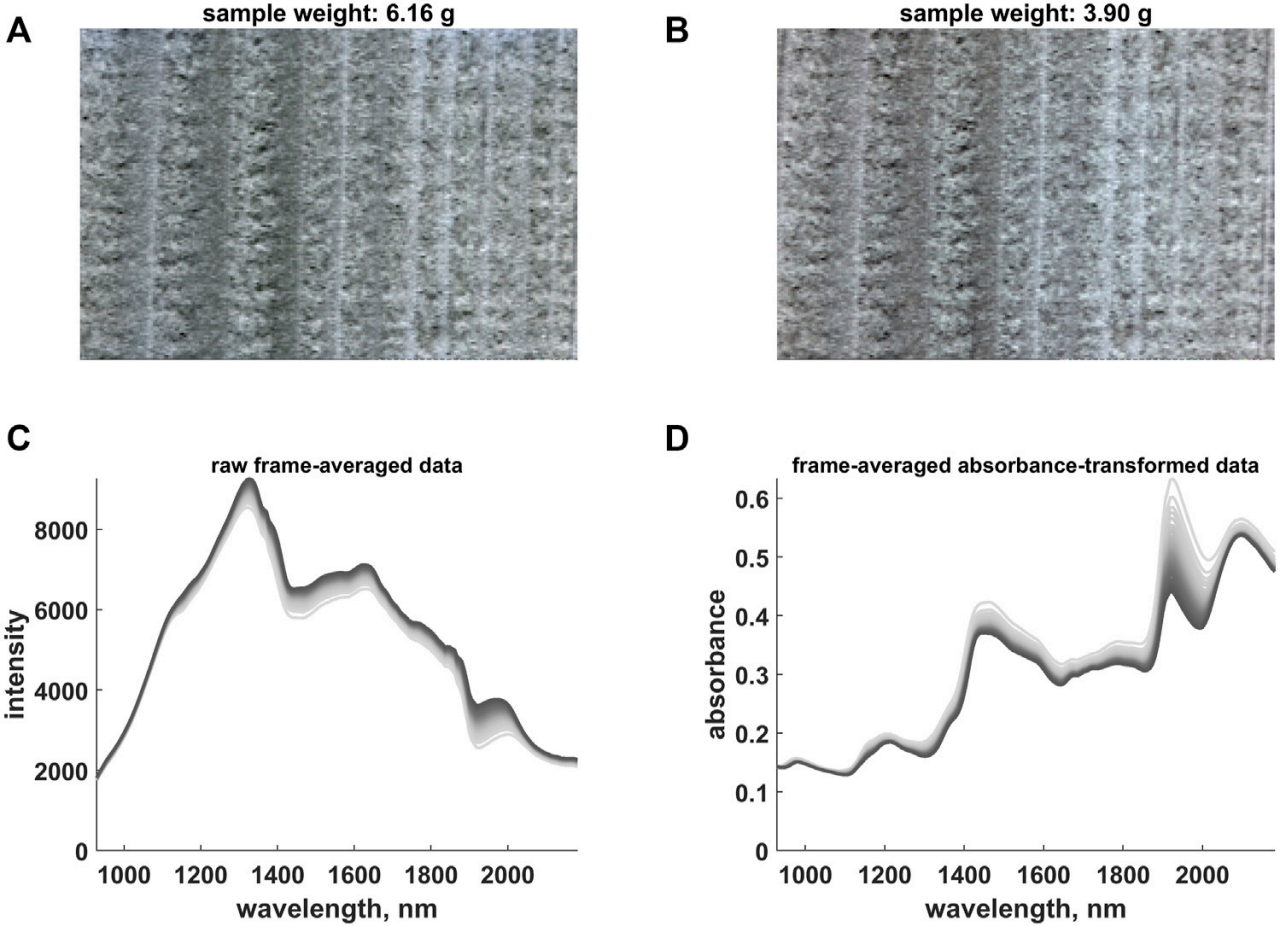
**(A)** False Red Green Blue (RGB) representation of the first hyperspectral video frame. **(B)** False RGB representation of the last hyperspectral video frame. **(C)** Raw frame-averaged intensity data. **(D)** Frame-averaged absorbance-transformed data. The colour gradient (from light to dark grey) follows the time evolution of the hyperspectral video. Notice that the absorbance values measured at 980, 1,138 and 1,302 nm were used to generate **(A)** and **(B)**.

As model system, a piece of wood of the species Norway Spruce (*Pincea abies*) was submerged in water and soaked for approximately 24 h. Thereafter, it was placed on a digital scale for tracking in real time the variation of its weight and its drying process was monitored by means of a hyperspectral line scan camera (Specim, Oulu, Finland) automatically capturing reflectance images between 930 and 2,200 nm. More specifically, the sample was scanned at regular time intervals, *i*.*e*., each time a decrease of around 0.05 g was observed (initial weight: 6.16 g—see Figure 2A; final weight: 3.90 g—see Figure 2B; total number of hyperspectral images: 42). The sample was illuminated by two halogen lamps positioned on the two sides of the hyperspectral device and never moved during the whole duration of the experiment. A region of interest of 150 × 225 pixels was segmented within each frame, which finally resulted in the generation of a four-dimensional dataset of size 150 × 225 × 42 × 200 (see also Section 1.3) and in a memory load of roughly 2.3 GB (double-precision floating-point format).

Although these data were already investigated before—Vitale et al. (2020b)—here, the key role of the linearisation of the instrumental response across space provided by the IDLE approach and its fundamental impact on the assessment and interpretation of the temporal variations of the water signal contributions will be explored.

## 4 Results and Discussion

**FIGURE 3:**
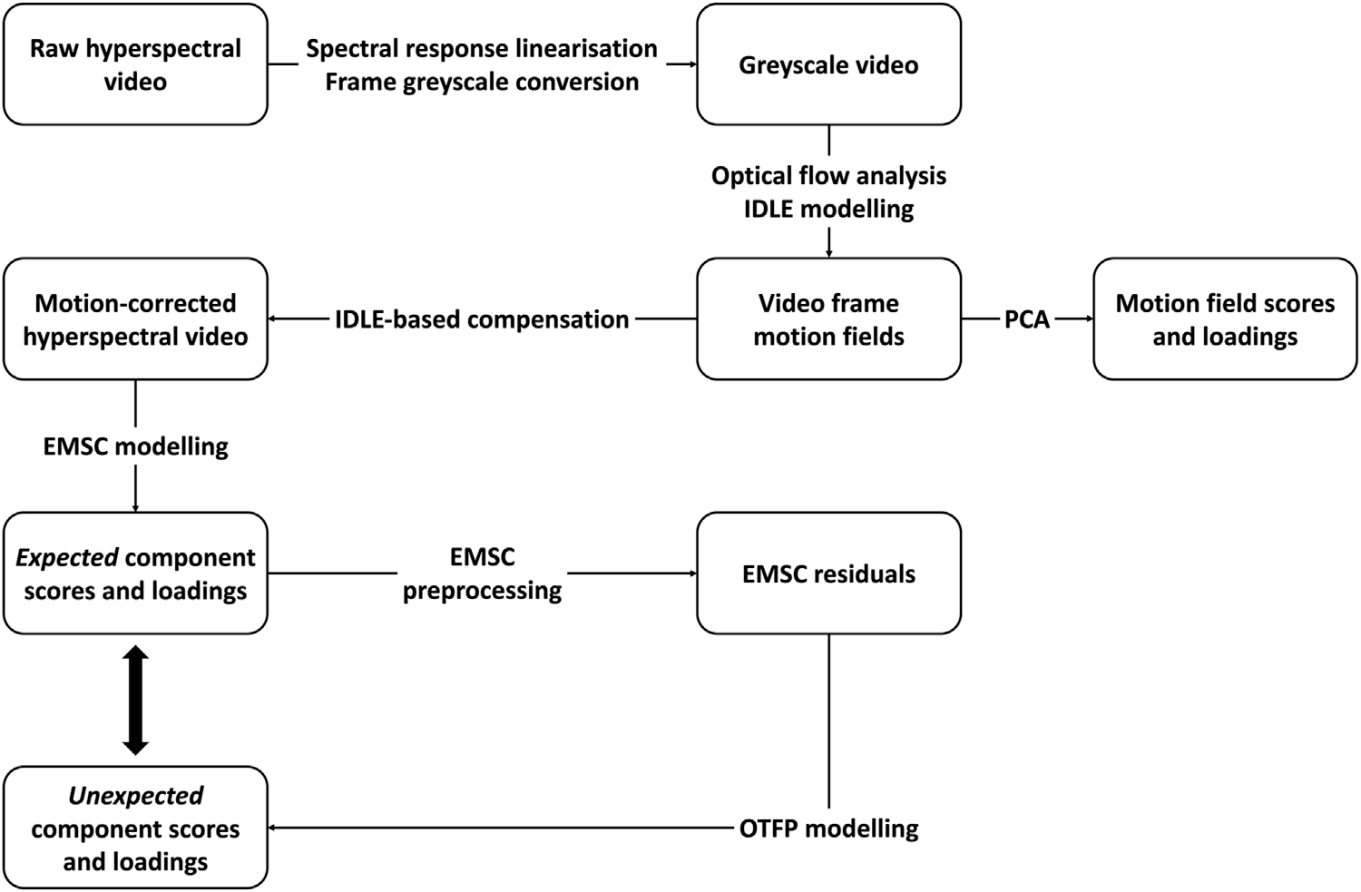
Schematic flowchart of the hyperspectral video processing and analysis framework proposed in this article.

A flowchart schematising the general hyperspectral video analysis framework proposed in this work is provided in Figure 3.

### 4.1 Spectral Response Linearisation and Frame Greyscale Conversion

In order to compensate the wavelength-dependent variations associated to the light source, the intensity values registered at each *j*th wavelength and at each *n*_*x*_ × *n*_*y*_-th pixel of the *k*th video frame, $I_{n_{x},n_{y},k,j}$ , were first converted into reflectance units as in :

$$R_{n_{x},n_{y},k,j}=\frac{\left(I_{n_{x},n_{y},k,j}-I_{n_{x},n_{y},j,d}\right)}{\left(I_{n_{x},n_{y},j,w}-I_{n_{x},n_{y},j,d}\right)}$$ (15)

with $I_{n_{x},n_{y},j,d}$ and $I_{n_{x},n_{y},j,w}$ the intensity recorded at the *j*th wavelength and at *n*_*x*_ × *n*_*y*_-th pixel for a dark reference and a white reference (a Spectralon sample), respectively. Thereafter, they were transformed into apparent absorbance (that is to say, linearised with respect to the chemical response) according to the following relation:

$$A_{n_{x},n_{y},k,j}=\log\left(\frac{1}{R_{n_{x},n_{y},k,j}}\right)=x_{n_{x},n_{y},k,j}$$ (16)

An example of raw and absorbance-converted spectral profiles is provided in Figure 2C,D, which highlight the presence of strong baseline variations probably caused by fluctuations in the illumination conditions or in the angular distribution of the reflected light. In order to minimise the bias that such fluctuations (unrelated to sample motions^2^) may induce in the IDLE-based quantification and compensation, an additional two-step pretreatment procedure was executed prior to the successive data processing stage:

1. the spectra associated to the pixels of each video frame were pretreated according to an EMSC model similar to the one in Eq. 8 and encompassing the profiles of two known components: dry wood^3^ (reference) and pure water^4^. **W**_EMSC_ was set equal to the identity matrix. More specifically, the correction performed for the *n*_*x*_ × *n*_*y*_-th pixel of the *k*th video frame can be expressed as:

$$x_{n_{x},n_{y},k}^{\text{p}}=\frac{\left(x_{n_{x},n_{y},k}-a_{k}1-d_{k}f-g_{k}f^{2}\right)}{b_{k}}-h_{k,water}s_{\text{water}}$$ (17)

with *a*_*k*_, *d*_*k*_, *g*_*k*_, *b*_*k*_ and *h*_*k*,water_ being estimated as in Eq. (9) from the *k*th frame mean spectrum;

2. at each time point, a grey-scale image, **I**_*k*_, was then obtained by averaging, for every pixel, the resulting absorbance values at 1,024, 1,195 and 1,309 nm (at these wavelengths, the frame-averaged spectra in Figure 2D exhibited the lowest standard deviation). In order to compensate dissimilarities among the intensity cumulative histograms of the various snapshots, these final estimates were ultimately level- and range-adjusted as:

$$I_{k}^{\text{p}}=\left(I_{k}-{\tilde{I}}_{k}\right)\frac{{RMS}_{\text{ref}}}{{RMS}_{k}}+{\tilde{I}}_{\text{ref}}$$ (18)

where ${\tilde{I}}_{k}$ and ${\tilde{I}}_{\text{ref}}$ are the median intensity values within the *k*th and the reference frame (n. 42—sample weight: 3.90 g), respectively, while *RMS*_ref_ and *RMS*_*k*_ represent the root-median-squared deviation of the pixel intensities in **I**_ref_ and **I**_*k*_ from their corresponding median values.

### 4.2 IDLE Modelling

**FIGURE 4:**
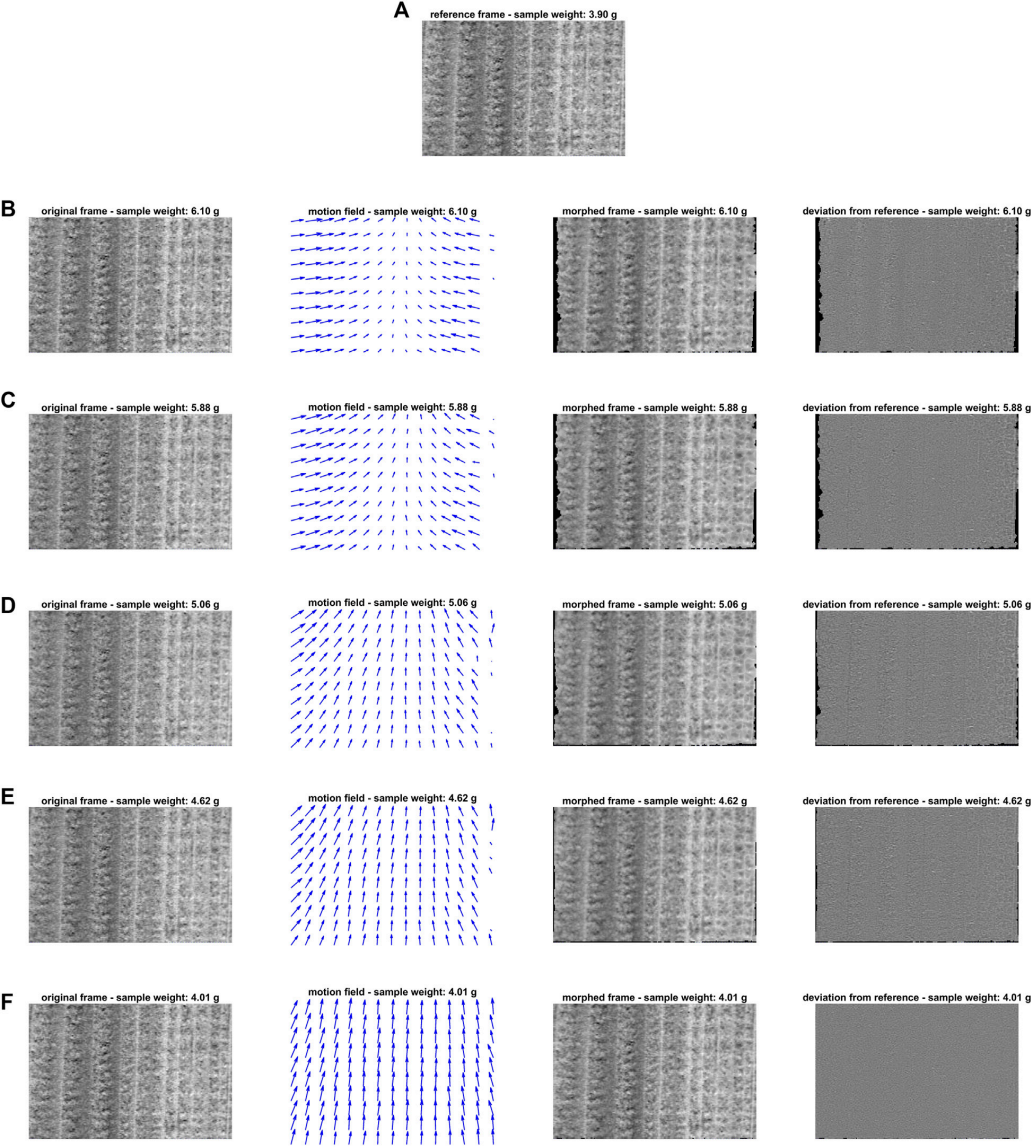
IDLE modelling: **(A)** displays the reference video frame; **(B-F)** contain (from left to right) the representation of five different snapshots collected over the entire duration of the monitoring experiment (n. 2—sample weight: 6.10 g; n. 6—sample weight: 5.88 g; n. 21—sample weight: 5.06 g; n. 29—sample weight: 4.62 g; n. 40—sample weight: 4.01 g), of the motion fields yielded by their optical flow analysis highlighting how individual pixels shifted compared to the reference image, of the motion-compensated frames morphed in order to mimic the target one and of the intensity deviations between the motion-compensated and the reference snapshots. Notice that IDLE was applied to grey-scale images, obtained by averaging the preprocessed absorbance values (see Section 4.1) at 1,024, 1,195 and 1,309 nm, respectively.

The level- and range-corrected grey-scale images output by the algorithmic procedure outlined in Section 4.1 were then subjected to IDLE modelling. Figure 4 summarises the outcomes of the motion estimation-compensation step: Figure 4A displays the reference video frame, while, for the sake of illustration, Figure 4B–F contain (from left to right) the representation of five other snapshots collected over the entire duration of the monitoring experiment, of the motion fields yielded by their optical flow analysis highlighting how individual pixels shifted compared to the reference image, of the motion-compensated frames morphed in order to mimic the target one and of the intensity deviations between the motion-compensated and the reference snapshots. As one can clearly see, except for minor edge artefacts, the aforementioned motion fields show how the wood sample horizontally squeezed as it dried and how such horizontal movements significantly decreased at the latest stage of the video recording (*i*.*e*., when low amounts of water were present in the pores between wood fibres and compression finally slowed down or stopped). This is also corroborated by the gradual reduction of the number of pixels whose motions could not be properly estimated by IDLE (see the black areas surrounding the motion-compensated frames) because of their relatively large displacement with respect to snapshot n. 42^5^. Notice that these pixels did not undergo EMSC-OTFP processing. Moreover, minimal intensity-deviation-from-target values were observed after image compensation, confirming that wood spatial variations were successfully corrected for.

**FIGURE 5:**
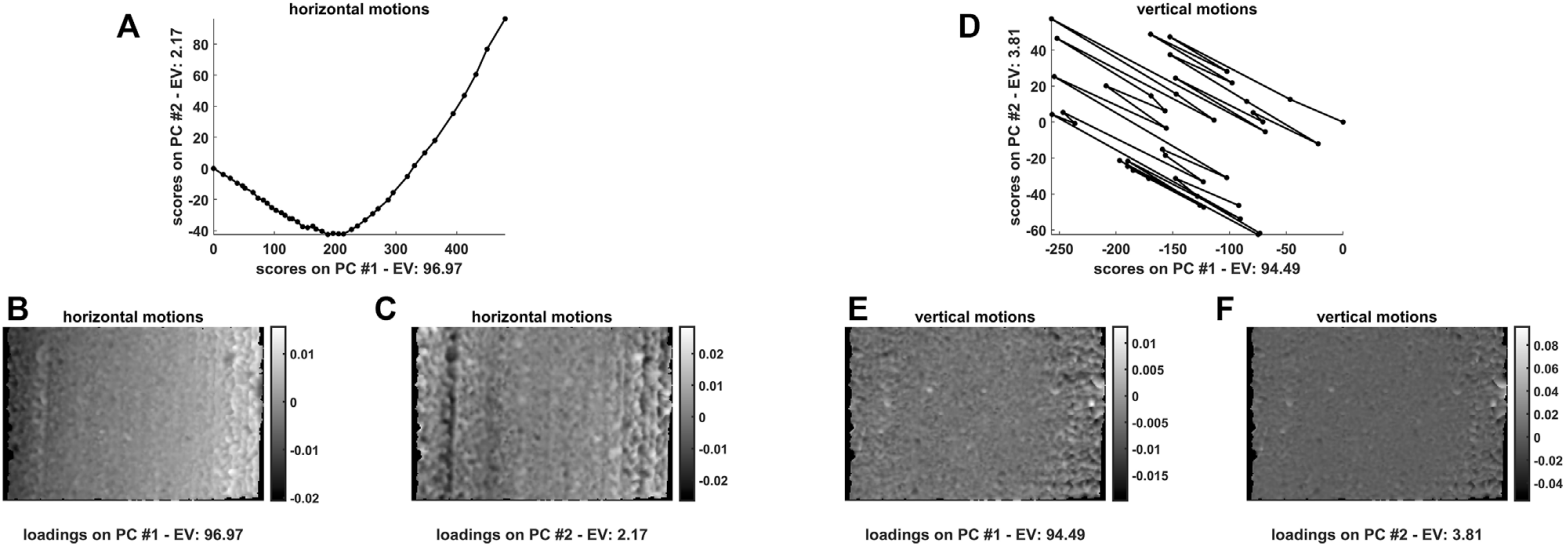
First and second principal component scores and loadings resulting from a bilinear decomposition of the **(A-C)** horizontal and **(D-F)** vertical motions quantified for the 42 hyperspectral video frames at hand. The black solid line follows the temporal evolution of the experiment. The reference snapshot (n. 42—the last one of the sequence) is easily recognisable as it exhibits scores coordinates equal to $\left[0,0\right]$ (*i*.*e*., no motions were estimated for it). The black areas around the loadings images contain pixels excluded from the computational procedure as they underwent an excessively large displacement with respect to frame n. 42. *EV* stands for *Explained Variance*.

In order to get additional insights into the nature of such spatial variations along time, the quantified horizontal and vertical motions—retrieved from all the calculated motion fields and concatenated as detailed in Martens (2015)—were analysed by PCA. The resulting temporal scores and spatial loadings are graphed in Figure 5.

While vertical shifts appear to follow a random trend (see Figure 5D) and might be looked at as mainly due to sideways camera or measurement stage bumps (loadings values are also more or less homogeneously distributed all over the inspected field of view—see Figures 5A,E,F) a smoother and more structured evolution was found for the horizontal ones, which further substantiates what stated before about wood squeezing. Horizontal motion scores (see Figure 5A) seem to point out the occurrence of a two-phase process during which compression initially proceeds faster and finally decelerates. Horizontal motion loadings along the first principal component (see Figure 5B) emphasise the differences between the movements of the pixels of the left and the right side of the image, while those along the second principal component (see Figure 5C) permit to distinguish the distinct behaviour of lateral and central pixels.

### 4.3 EMSC Modelling

If on the one hand the IDLE approach is capable of quantifying and compensating the movements of a sample observed throughout a hyperspectral video (thus, enhancing the spatial linearity of the instrumental response), on the other hand the combined use of EMSC and the OTFP can enable the identification and retrieval of the most meaningful sources of information from the time series of resulting motion-free hyperspectral images.

**FIGURE 6:**
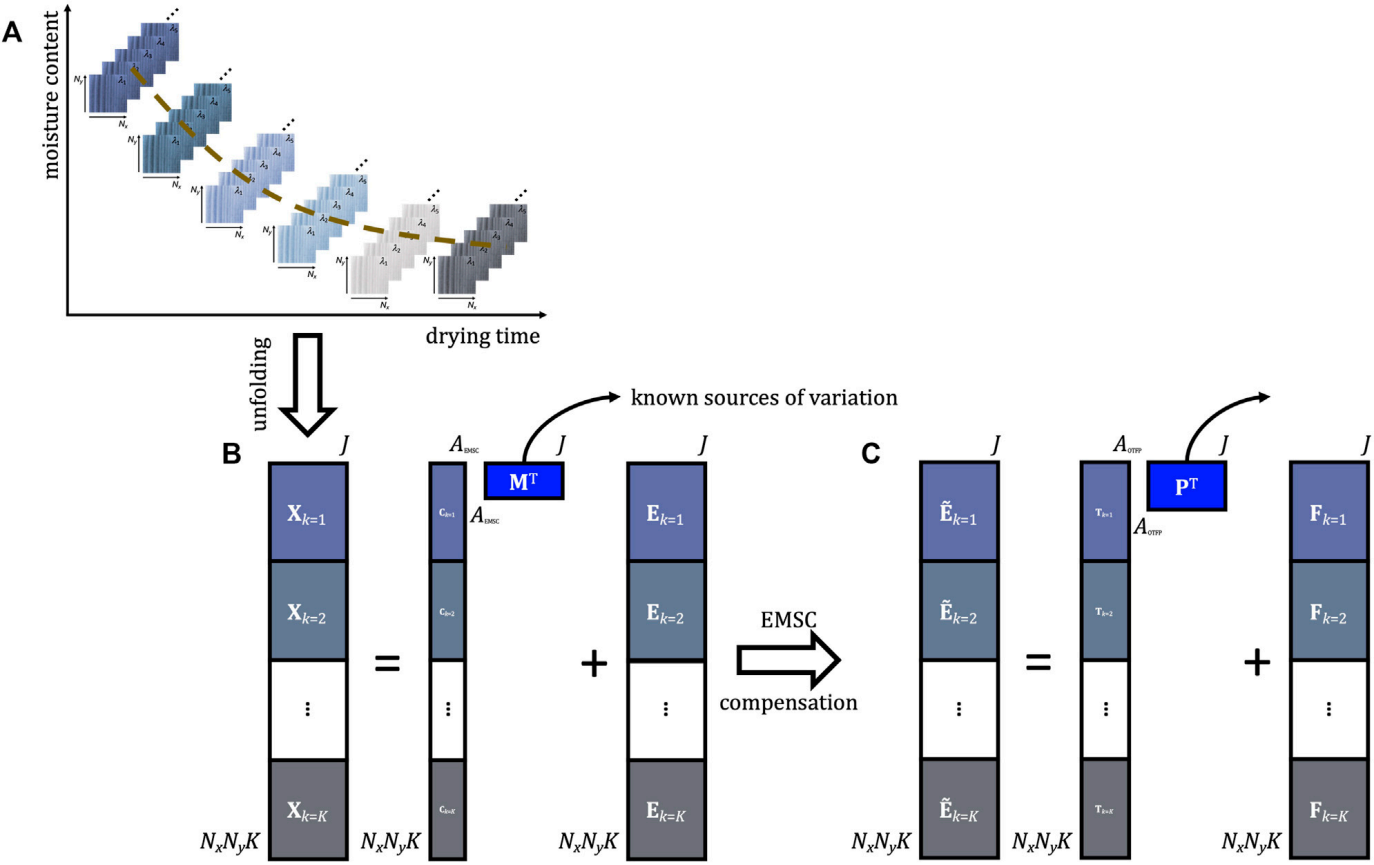
Schematic representation of the EMSC-OTFP analysis pipeline. **(A)** Example of hyperspectral video data structure. **(B)** EMSC modelling. **(C)** OTFP modelling.

The EMSC-OTFP analysis pipeline is schematically outlined in Figure 6.

Both EMSC and the OTFP are bilinear modelling techniques that can be utilised in an adaptive- or recursive-like way without requiring entire raw datasets to be kept in memory. The main difference between them regards their respective subspace definition. The matrix **M** (see Eq. 9 and Figure 6B), in fact, is manually constructed by the user based on *a priori* knowledge about the system or the sample under study, which renders EMSC an ideal methodology for extracting and describing expected variation patterns evolving during the progression of a hyperspectral video. On the other hand, **P** (see Eq. 14 and Figure 6C) is automatically learnt by the OTFP algorithm which gradually discovers (in real time) all the sources of systematic variation underlying the data at hand. Consequently, applying sequentially 1) EMSC to the (unfolded) motion-corrected data and 2) the OTFP to the resulting EMSC residuals yields two additive models accounting for both known and unknown phenomena driving the generation mechanism of hyperspectral videos and providing a detailed global overview of the captured dynamic scene.

**FIGURE 7:**
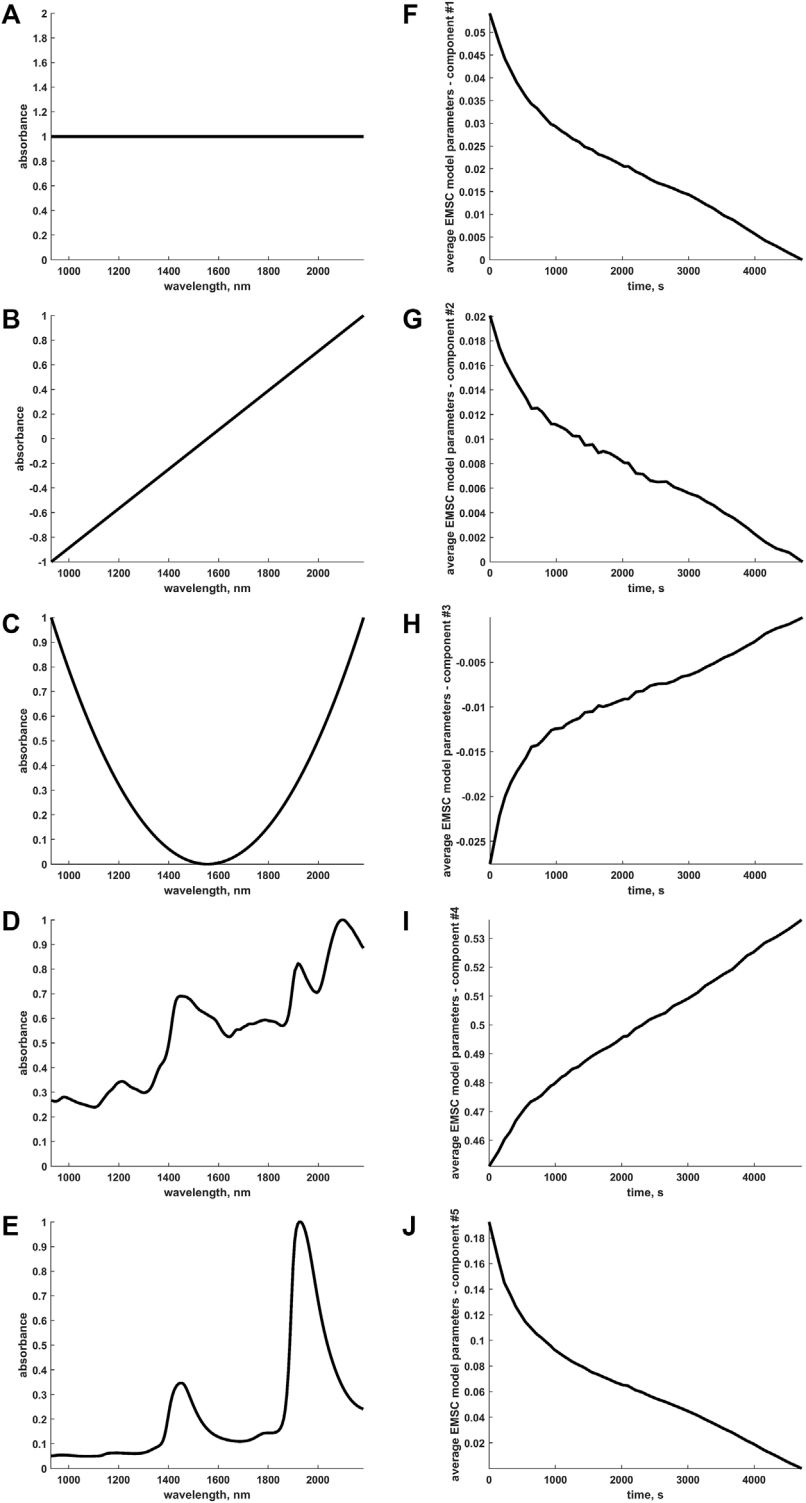
**(A-E)** Characteristic (absolute max-normalised) spectral profiles submitted to the EMSC computational procedure. The first three represent a typical choice for EMSC modelling as they allow baseline offset, slope and quadratic curvature to be estimated for all the spectra or pixels of the hyperspectral video and compensated before the subsequent On-The-Fly Processing. The last two correspond to the spectra of dry wood and pure water, the two main constituents underlying the specific scene at hand. **(F-J)** Time evolution of the frame-averaged EMSC coefficients (rescaled to compensate the aforementioned absolute max-normalisation) associated to the sources of variation explained by the spectral profiles in **(A-E)**.

Here, in a first step, the five profiles in Figure 7A–E were input to the EMSC algorithmic procedure: as also briefly outlined before, the first three constitute a standard choice for EMSC modelling as they permit to estimate and compensate baseline offset, slope and quadratic curvature for all the pixels of the hyperspectral video before the subsequent application of the OTFP. The last two profiles, instead, correspond to the spectra of dry wood (reference) and pure water, the two major constituents of the specific scene at hand. Representing the time trend of the coefficients yielded for each one of these expected sources of data variability (averaged across all the pixels within every original video frame after motions were compensated, see Figures 7F–J) is a simple and immediate way to visualise and assess the information returned by EMSC and somehow characterise the dynamic evolution of known variability patterns during wood drying. From such graphs, one can easily observe that most of the modelled wood features change quite rapidly within the first stage of the drying process. This may be due to the residual presence of a thin liquid water film on the surface of the wood sample at the beginning of the hyperspectral monitoring, which could have cloaked its spectral properties. Figure 7J also highlights that moisture loss was still proceeding when the experiment was interrupted. Conversely, regarding the wood contribution itself, an approximately constant increasing trend over time was observed. This behaviour accurately reflects the chemical nature of the sample drying which might have been clearly unveiled here because its continuous physical contractions were directly and explicitly accounted for, significantly reducing the spatial complexity of the considered video data. It goes without saying, then, that exploiting simultaneously both spectral and spatial information encoded in hyperspectral videos can significantly enhance the comprehension and understanding of the physico-chemical phenomena behind complex real-world systems.

### 4.4 OTFP Modelling

**FIGURE 8:**
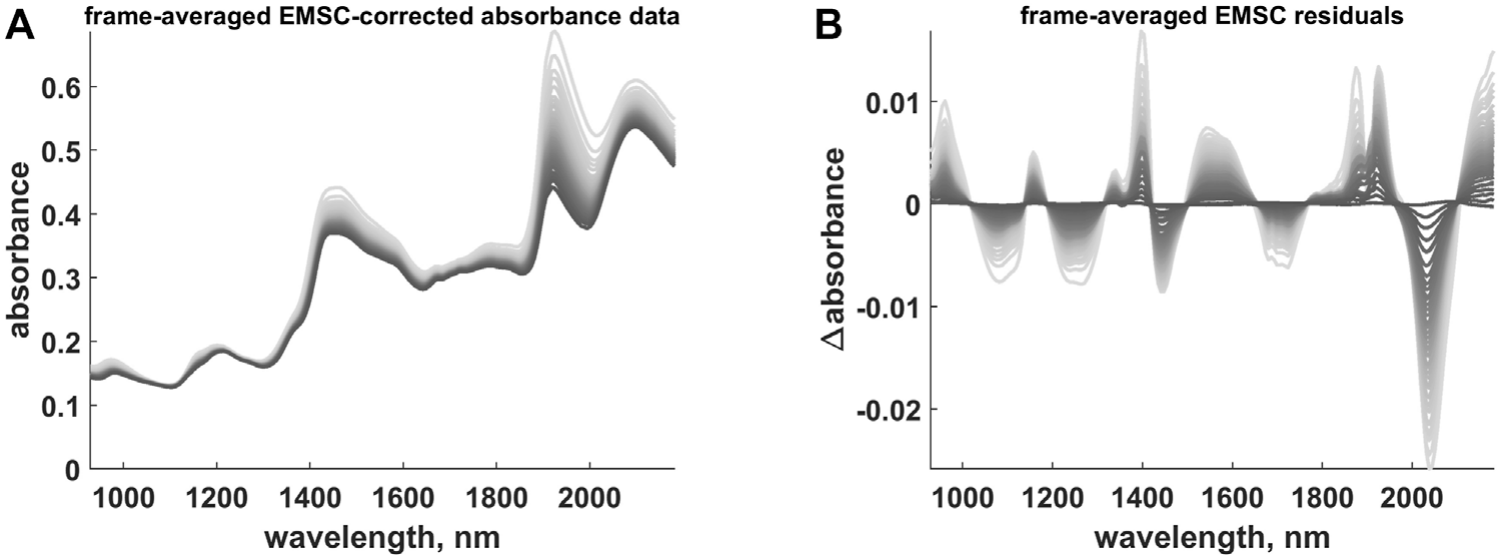
**(A)** Frame-averaged EMSC-corrected absorbance data. **(B)** Frame-averaged EMSC residual profiles. The colour gradient (from light to dark grey) follows the time evolution of the hyperspectral video.

After the EMSC compensation (see Figure 8A), the resulting residual profiles (see Figure 8B) were submitted to the OTFP for automatically retrieving all the systematic sources of variation left unmodelled by the first data analysis steps^6^.

**FIGURE 9:**
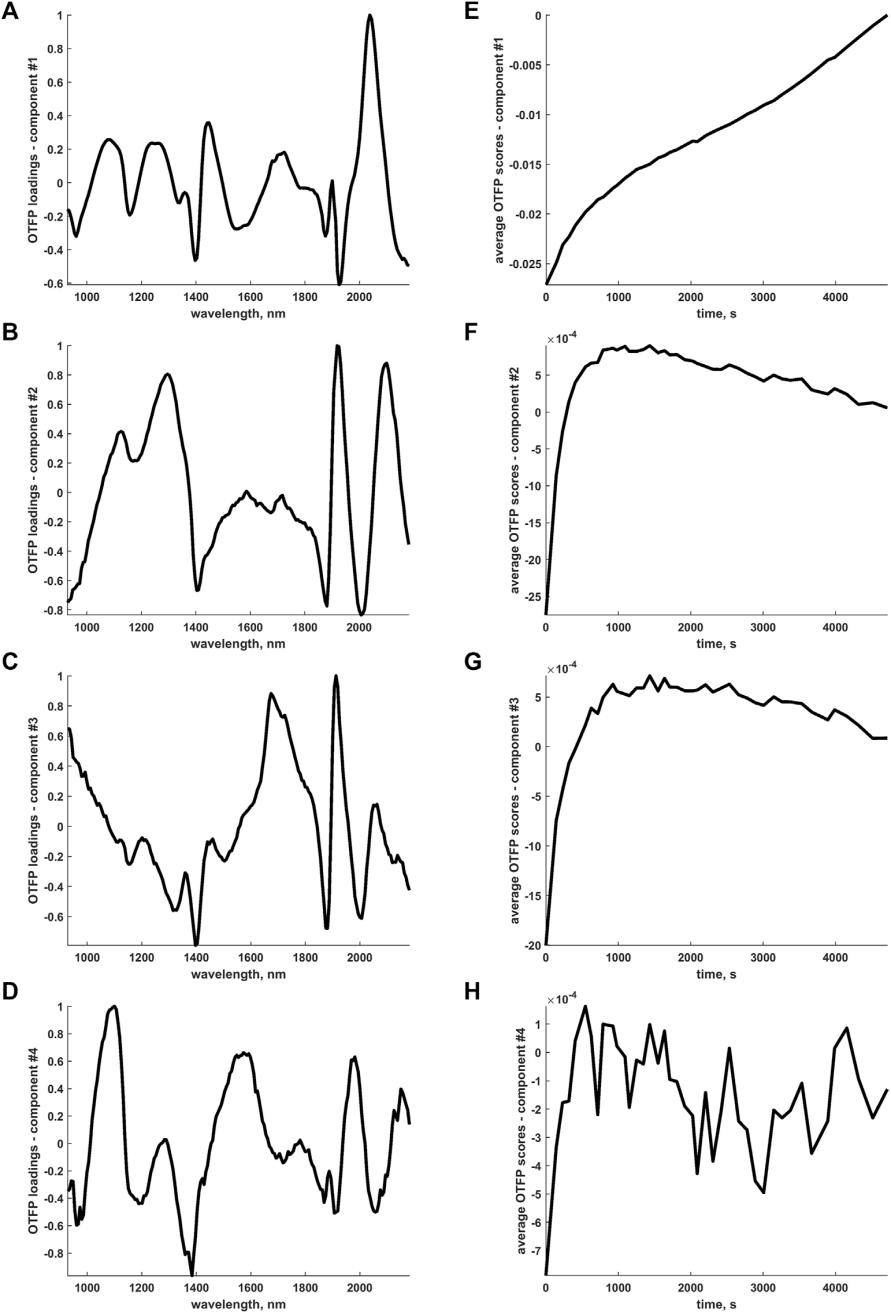
**(A-D)** Pseudo-spectral (absolute max-normalised) loadings profiles retrieved by the OTFP computational procedure. **(E-H)** Time evolution of the frame-averaged OTFP scores (rescaled to compensate the aforementioned absolute max-normalisation) associated to the sources of variation explained by the loadings profiles in **(A-D)**.

Even if the interpretation of the OTFP output may seem more complicated due to the fact that the OTFP subspace features PCA-like orthogonal bases, smooth and rather well-defined time trends were found for the frame-averaged OTFP coefficients or scores (see Figure 9E–H). Such time trends highlight the existence of at least two structured phases in the process of wood drying. Consider, for example, Figure 9F: an initial fast transition from negative to positive scores values can be observed followed by a smoother descendant evolution approximately plateauing at around 0. Given also that most of the OTFP loadings profiles in Figure 9A–D show large contributions associated to the main water absorption regions, one can reasonably envision the occurrence of more complex phenomena directly related to the thermodynamic state of water itself (*i*.*e*., free or bound).

### 4.5 Data Reconstruction and Postprocessing

**FIGURE 10:**
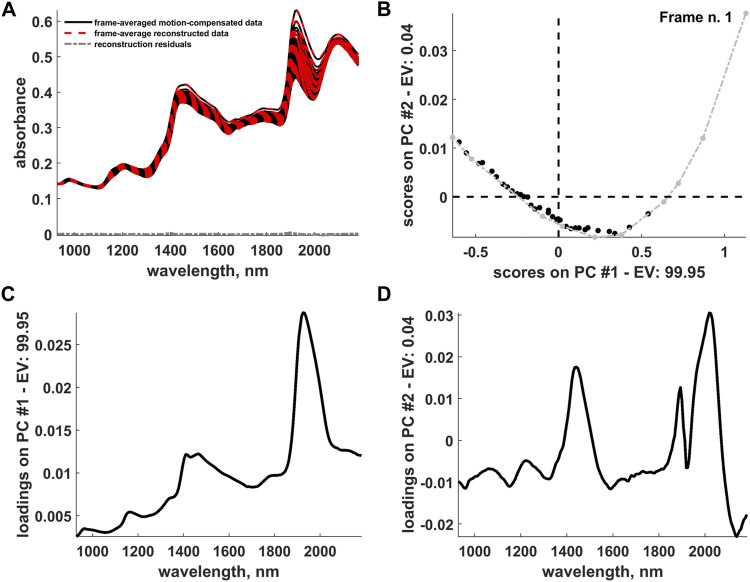
**(A)** Representation of the frame-averaged motion-compensated data, frame-averaged data reconstructed after the IDLE, EMSC and OTFP analysis and reconstruction residuals. **(B)** Two-dimensional scores plot resulting from a PCA decomposition of the (pathlength-corrected) frame-averaged reconstructed data. Archetypal frames are highlighted in light grey and connected by a dashed-dotted grey line. The evolution of the scores from right to left follows the hyperspectral video progression from its beginning to its end. **(C)** First and **(D)** second component loadings yielded by the aforementioned PCA decomposition. *PC* and *EV* stand for *Principal Component* and *Explained Variance*, respectively.

**FIGURE 11:**
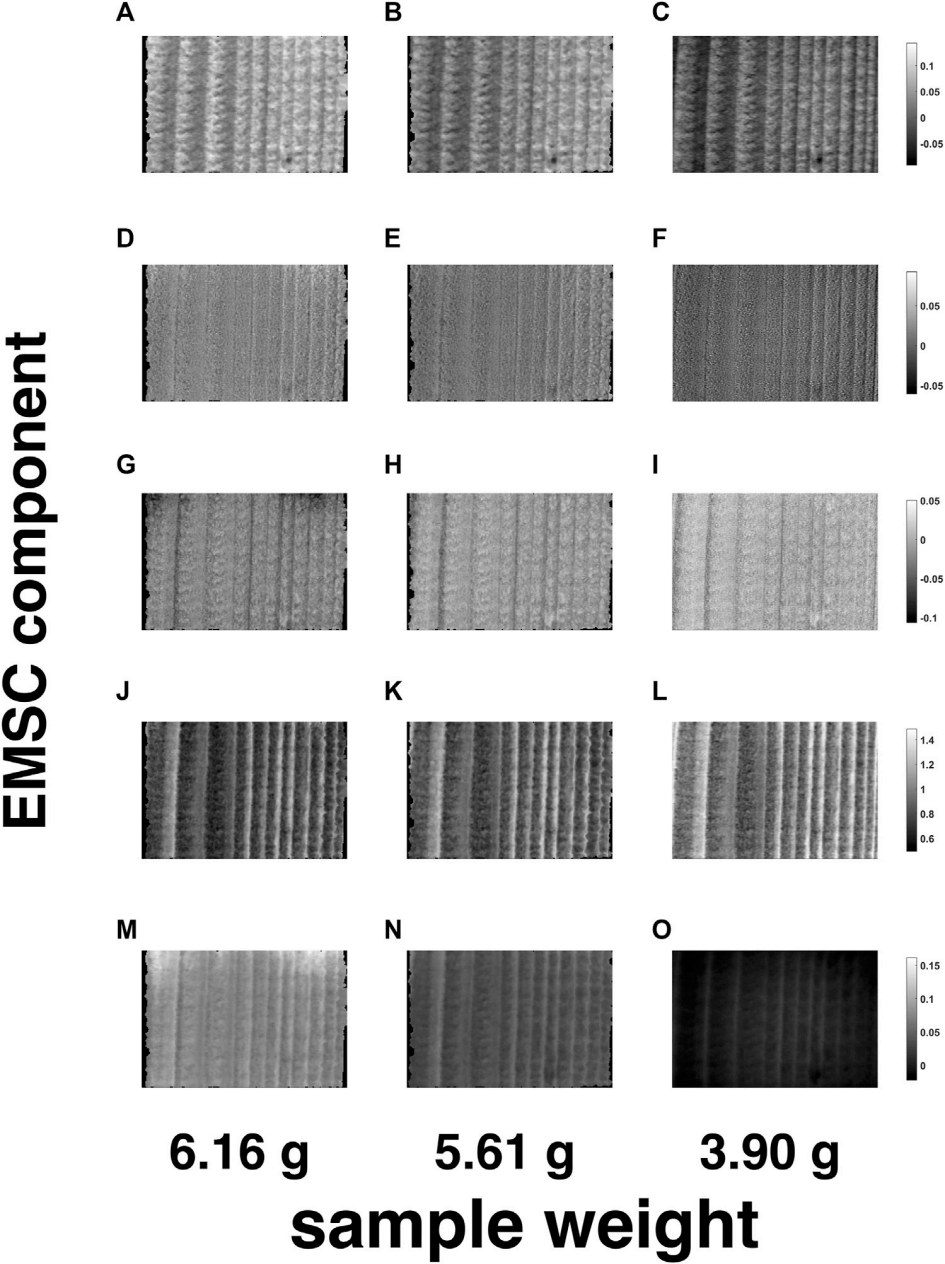
Spatial representation of the EMSC coefficients related to the EMSC components n. 1—**(A-C)**—n. 2—**(D-F)**—n. 3—**(G-I)**—n. 4—**(J-L)**—and n. 5—**(M-O)**—for three of the 10 archetypal frames highlighted in Figure 10B. The black areas around the loadings images contain pixels excluded from the computational procedure.

**FIGURE 12:**
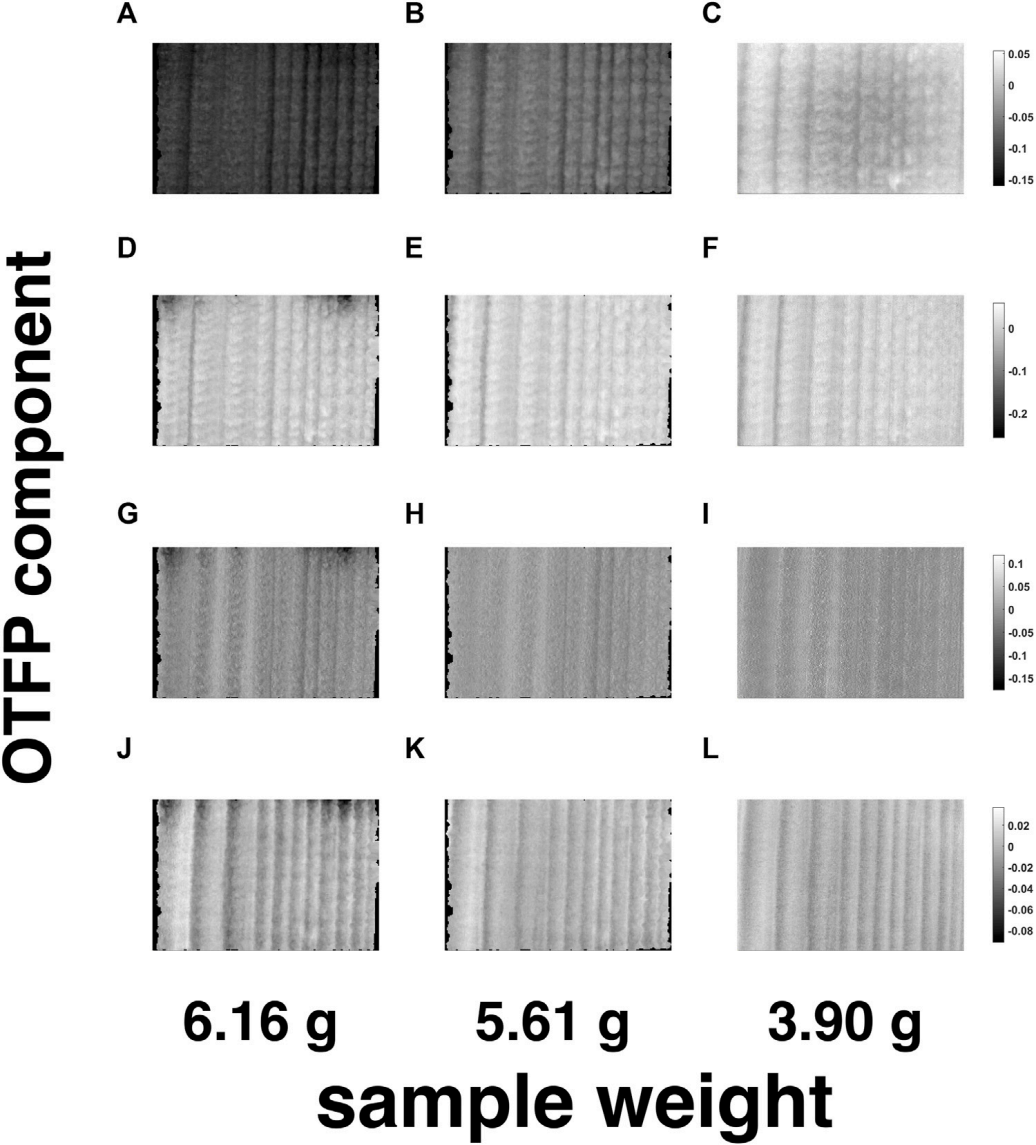
Spatial representation of the OTFP scores related to the OTFP factors n. 1—**(A-C)**—n. 2—**(D-F)**—n. 3—**(G-I)**—n. 4—**(J-L)**—for three of the 10 archetypal frames highlighted in Figure 10B. The black areas around the loadings images contain pixels excluded from the computational procedure.

For a tentative exploration of the thermodynamic phenomena mentioned in Section 4.4, the pathlength-corrected absorbance spectra, obtained by reconstructing and averaging the 42 motion-compensated hyperspectral video frames after EMSC and OTFP processing (see Figure 10A), were decomposed by standard PCA and graphed in the scores plot in Figure 10B. This plot clearly highlights the occurrence of a two-phase transition process during wood drying affecting mainly the water bands of such NIR spectra (see the loadings in Figures 10C,D) and characterised by 10 archetypal time instants (see the grey dots in Figure 10B)—Ruckebusch et al. (2020). Figures 11, 12 provide an illustration of the distribution of the EMSC coefficients and the OTFP scores over the surface of the wood sample at three of these time instants. This representation allows assessing the aforementioned transition process at a spatial level: overall, the coefficient spatial distribution seems to get smoother as the experiment evolves, which might be explained in the light of the continuous migration/diffusion of water molecules through the pores of the wood specimen (see, *e*.*g*., Figure 12D–F). However, all these aspects will be investigated in future research also by means of more rational subspace axis rotations—performed, for instance, by varimax, Kaiser (1958), Independent Component Analysis (ICA), Comon (1994); Hyvärinen et al. (2001), or MCR-ALS—aimed at optimising the meaningfulness of the OTFP factors from a physico-chemical perspective.

## 5 Conclusion

Hyperspectral videos generate a lot of informative data. However, these data require efficient mathematical modelling for being reliable, understandable and quantitatively interpretable. Here, a general framework by which hyperspectral videos can be analysed was proposed. The three computational steps of this framework result in a compact multi-domain hybrid subspace modelling approach, involving spatial, spectral and temporal parametrisation of both known and unknown chemical and physical phenomena underlying the studied systems. IDLE permits to characterise and compensate the complex motions that the investigated objects may undergo over the measurement time. EMSC is capable of providing a simple mathematical description of a range of phenomena (and of their temporal evolution) that operators expect or presume *a priori* to be occurring over the duration of the hyperspectral video recording. Finally, the OTFP compresses and summarises all the information related to unknown or unexpected events which may happen during the progression of the data collection. In other words, one can look at the combination of these three different methodologies as an algorithmic extension of how human beings observe reality: the eyes capture spatial changes in the external environment and submit particular signals to the brain that afterwards processes them distinguishing between what was somehow forecastable in advance (based on past experiences) and what is completely new and unforeseen. In this regard, rather than the individual application of the aforementioned techniques (some of which are already well-established in the field of chemometrics), it is their fusion into a comprehensive algorithmic architecture for the global assessment and interpretation of time-series of high-dimensional hyperspectral images to be innovative and unprecedented.

The sequential IDLE-EMSC-OTFP hybrid framework presented here rests on a combination of targeted and non-targeted data modelling of both known and unknown variation sources. In contrast to classical subspace decomposition strategies (*e*.*g*., PCA, PLS, MCR-ALS, NNMF and ICA), it enables the description not only of additive spectral response variations, but also of multiplicative ones (like physical structure effects on the optical pathlength) and hard and soft shape changes (due, for example, to sample repositioning and/or shrinkage).

Moreover, differently from machine learning methods based on Artificial Neural Networks (ANN)—Gasteiger and Zupan (1993)—and Convolutional Neural Networks (CNN)—Gu et al. (2018)—the IDLE-EMSC-OTFP modelling approach yields a strong dimensionality reduction of torrents of input data and results graphically interpretable in their compressed state, revealing how spectral properties, spatial patterns and temporal dynamics are strictly intertwined into unified variation components, whose assessment and interpretation might provide fundamental insights into underlying chemical, physical and instrumental causalities. In the future, relying on a trilinear rather than bilinear OTFP model structure—exploiting, for instance, the principles of Parallel Factor Analysis (PARAFAC), Harshman (1970); Carroll and Chang (1970); Bro (1997)—may enhance this process further.

These conclusions are substantiated and thoroughly corroborated by the outcomes reported in this article. In fact:

1. motion estimation-compensation by spatiotemporal IDLE modelling allowed shrinkage induced by wood drying to be modelled and corrected for, reducing the spatial complexity of the hyperspectral imaging data;
2. EMSC preprocessing permitted a simpler spectral modelling by detecting and disentangling light absorption/light scattering-related variation patterns and their respective evolution over time;
3. the continuous data-driven bilinear subspace decomposition returned by the OTFP enabled the study of the dynamics of the various physical and chemical variations left unmodelled in the stream of hyperspectral residuals after the previous two steps.

In the light of all this and considering its computational efficiency when massive (potentially ever-lasting) flows of multi-channel measurements are handled, the developed approach could have an enormous impact also within the more general context of BIG DATA.

## Data Availability

Data are available under request. Inquiries can be directed to the corresponding author.

## Footnotes

1 If $\mathrm{diag}\left(W_{\text{EMSC}}\right)=1$ , the parameter estimation is carried out by OLS.

2 For example, those due to water diffusion.

3 Calculated as the average profile of the last video frame.

4 Measured in reflectance mode by a Nicolet 6700 FT-NIR instrument (Thermo Scientific Inc., Madison, WI, United States) at the same nominal resolution and within the same spectral range as for the hyperspectral video data dealt with in this study and, then, converted into absorbance units.

5 In fact, the higher the time difference between frames, the larger the distance that the pixels at the borders of these frames covered due to wood squeezing.

6 4 OTFP components were required to explain around 80% of the EMSC residual variance.
