# Corrigendum: Hyperspectral Video Analysis by Motion and Intensity Preprocessing and Subspace Autoencoding

**DOI:** 10.3389/fchem.2022.926330

**Published:** 2022-05-18

**Authors:** Raffaele Vitale, Cyril Ruckebusch, Ingunn Burud, Harald Martens

**Affiliations:** ^1^ Univ. Lille, CNRS, LASIRE (UMR 8516), Laboratoire Avancé de Spectroscopie pour les Interactions, la Réactivité et l’Environnement, Lille, France; ^2^ Faculty of Science and Technology, Norwegian University of Life Sciences, Oslo, Norway; ^3^ Idletechs AS, Trondheim, Norway; ^4^ Department of Engineering Cybernetics, Norwegian University of Science and Technology, Trondheim, Norway

**Keywords:** hyperspectral videos, motion compensation, IDLE modelling, light scattering, light absorption, extended multiplicative signal correction, on-the-fly processing, BIG measurement DATA

In the original article, there was an error in Figure 10B and its caption on page 13. The initial frame of the hyperspectral video at hand was mistakenly labelled. The correct version of Figure 10B with its new caption appears below.

**FIGURE 10:**
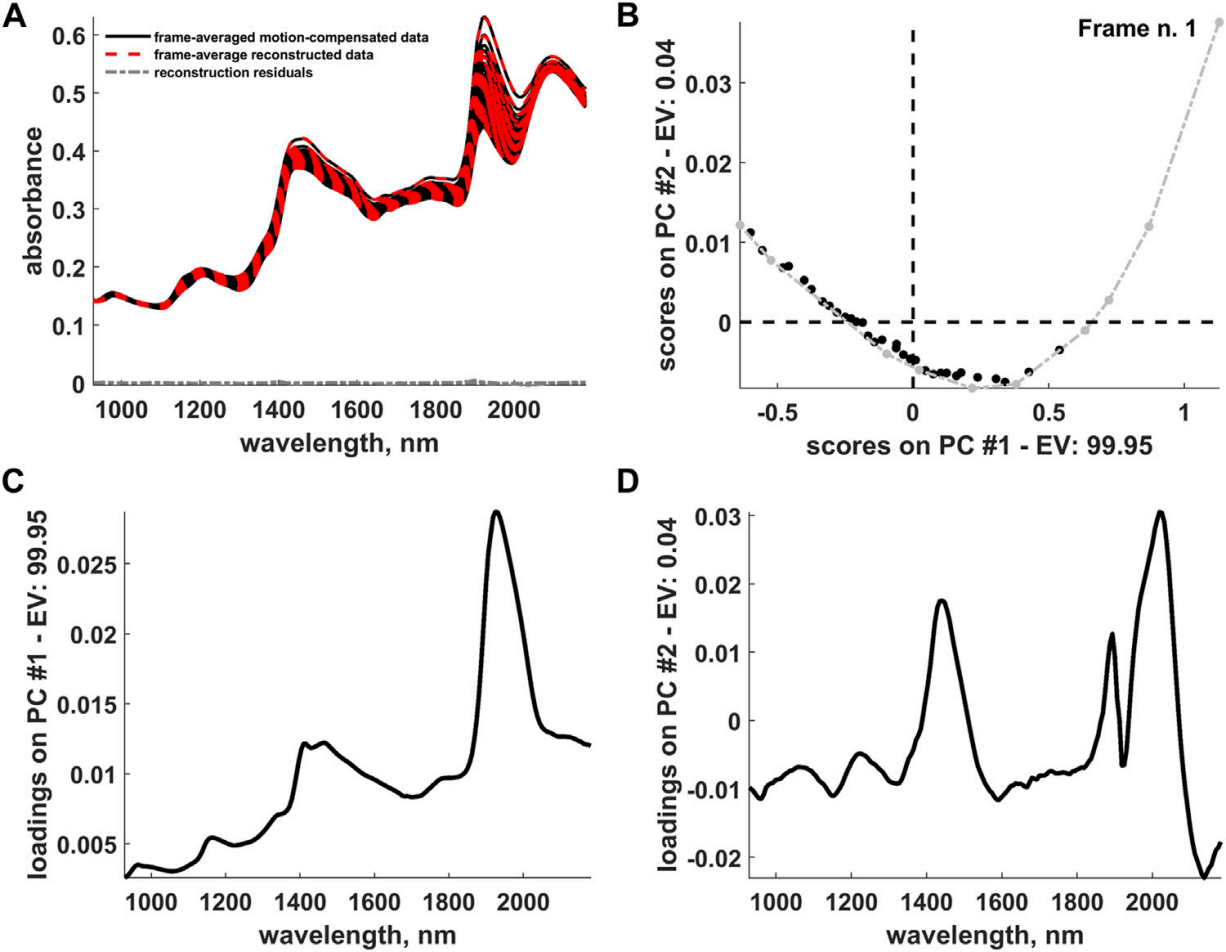
**(A)** Representation of the frame-averaged motion-compensated data, frame-averaged data reconstructed after the IDLE, EMSC and OTFP analysis and reconstruction residuals. **(B)** Two-dimensional scores plot resulting from a PCA decomposition of the (pathlength-corrected) frame-averaged reconstructed data. Archetypal frames are highlighted in light grey and connected by a dashed-dotted grey line. The evolution of the scores from right to left follows the hyperspectral video progression from its beginning to its end. **(C)** First and **(D)** second component loadings yielded by the aforementioned PCA decomposition. *PC* and *EV* stand for *Principal Component* and *Explained Variance*, respectively.

The authors apologize for this error and state that this does not change the scientific conclusions of the manuscript in any way.

